# Resurgence of Respiratory Virus after Relaxation of COVID-19 Containment Measures: A Real-World Data Study from a Regional Hospital of Italy

**DOI:** 10.1155/2022/4915678

**Published:** 2022-11-25

**Authors:** Davide Treggiari, Chiara Piubelli, Fabio Formenti, Ronaldo Silva, Francesca Perandin

**Affiliations:** Department of Infectious Tropical Disease and Microbiology, IRCCS Sacro Cuore Don Calabria Hospital, Negrar di Valpolicella, Verona, Italy

## Abstract

The SARS-CoV-2 virus spread in the Northern Hemisphere during the 2019/2020 influenza seasons and it persisted in the 2021/2022 season. A cocirculation of SARS-CoV-2 and influenza viruses was expected in Italy during the winter seasons. This study aims to investigate the prevalence of influenza and respiratory syncytial viruses observed in a hospital in Verona Province, Italy hospital during these past three winter seasons and to compare our data with national and global surveillance reports on the transmission of respiratory viruses in the preceding decade. Our findings clearly demonstrated the extremely low prevalence of influenza virus among hospitalized patients and outpatients during the first two COVID-19 winter seasons, with a reemergence of respiratory syncytial virus in the late 2021. Containment measures may have played an important role in temporarily stopping the circulation of respiratory viruses, but after relaxation, in 2021, we experienced an unusual increase of respiratory syncytial viruses at the beginning of the winter season.

## 1. Introduction

Influenza is a contagious disease of the respiratory tract caused by human influenza virus types A, B (INF-A/B), or C. Influenza infections typically show seasonality, with epidemic transmission intensity between autumn and spring. These annual epidemics constitute an important public health problem with substantial morbidity and mortality, and they have a considerable economic impact [1]. Another common cause of lower respiratory tract infection is respiratory syncytial virus (RSV), that is classified into two types, RSV-A and -B. RSV is responsible for numerous hospitalizations of children in their first years of life, which add to the seasonal burden on health-care services [2]. To date, vaccines to prevent RSV disease or curative treatment options are not available.

The pandemic severe acute respiratory syndrome coronavirus 2 (SARS-CoV-2) spread in Italy and the rest of the Northern Hemisphere during the 2019-2020 winter season and continues to circulate even during the season 2020/21 and 2021/22. On January 31^st^ 2020, following the recommendations of WHO PHEIC [3], the Council of Ministers declared a state of health emergency due to the SARS-CoV-2 epidemic. To contain the epidemic transmission of SARS-CoV-2, Italy introduced nonpharmaceutical interventions (NPI), including social distancing, face mask, hands hygiene, and announced the first complete lockdown of social activities between March and May 2020.

The clinical presentation, transmission routes, and seasonality of SARS-CoV-2 resemble those of INF-A/B, and it can be difficult to distinguish the two infections. The Department of Infectious Tropical Diseases and Microbiology (DITM) at the IRCCS Sacro Cuore Don Calabria Hospital, therefore, implemented a diagnostic routine to test all persons for whom SARS-CoV-2 testing was requested, using a multiplex molecular assay that includes testing for SARS-CoV-2, INF-A/B, and RSV-A/B.

Here, we describe the diagnostic yield of INF and RSV testing in our catchment area since the start of the SARS-CoV-2 pandemic, and we compare our data with Italian and global virus surveillance data.

## 2. Methods

### 2.1. Study Design

We performed a retrospective observational analysis of INF-A/B and RSV-A/B molecular diagnostic test results obtained from patients who were referred to IRCCS Sacro Cuore Don Calabria Hospital (Italy). Nasopharyngeal swab samples processed by the routine diagnostics were collected during the seasons 2019/2020 (from week 46 in 2019 to week 12 in 2020), 2020/2021 (from week 47 in 2020 to week 17 in 2021) and 2021/2022 (from week 42 in 2021 to week 4 in 2022).

### 2.2. Study Population

The results for all diagnostic and screening INF-A/B and RSV-A/B tests (including all positive and negative results) were extracted from the IRCCS Laboratory Information Management System (LIMS) database. The retrieved data was cleaned by removing duplicate test results. Patients' age and sex were also collected.

### 2.3. Respiratory Virus Screening

Respiratory specimens were screened for respiratory viruses using GeneXpert Xpress Flu/RSV (Xpert; Cepheid, Sunnyvale, CA) during the season 2019/2020, and real-time PCR SARS-CoV-2/Flu/RSV Panel Kit (Anatolia Geneworks, Turkey, or Qiagen, NeuMoDx, USA) during 2020/2021 and 2021/2022 seasons. Influenza genotyping was performed by outsourcing at the regional referral laboratory of University of the Padova.

### 2.4. Statistical Analysis

Descriptive statistics were generated to define the study population. A comparison was made by an unpaired the two-tailed Student's *t*-test, *p* < 0.05 was defined as the level of significance. Graphpad (GraphPad Software, USA) was used to perform statistical analyses.

### 2.5. Ethics Statement

In accordance with EU General Data Protection Regulation (GDPR) 2016/679 and Good Clinical Practice standards, sensitive data were not collected. Each observation in the dataset was identified solely by an alphanumerical code not related to patient identification. Ethical clearance for the study protocol was obtained from the competent Ethics Committees of Verona and Vicenza provinces (Prot. 25099/2021).

## 3. Results

### 3.1. Demographic Distribution and Characteristics

All patients who underwent INF and RSV testing were included in this study. The demographic characteristics of all patients studied in the three influenza seasons are summarized in Table 1. Out of 32819 respiratory specimens, 167 (0.5%) were from season 2019/2020, 18112 (55.1%) from season 2020/2021 and 14540 (44.3%) from season 2021/2022. The age distribution of patients differed between seasons. During the 2019/2020 season, the age groups 0–12 and 60–99 years were best represented (37.7% and 43.7%, respectively). In the 2020/2021 season, these were patients aged 19–59 years (56.3%) and 60–99 (34.8%). In the 2021/2022 season, the age group 19–59 years comprised 49.2%, and 60–99 years 33.1% of the study population.

### 3.2. Prevalence of INF-A/B

**Figure 1:**
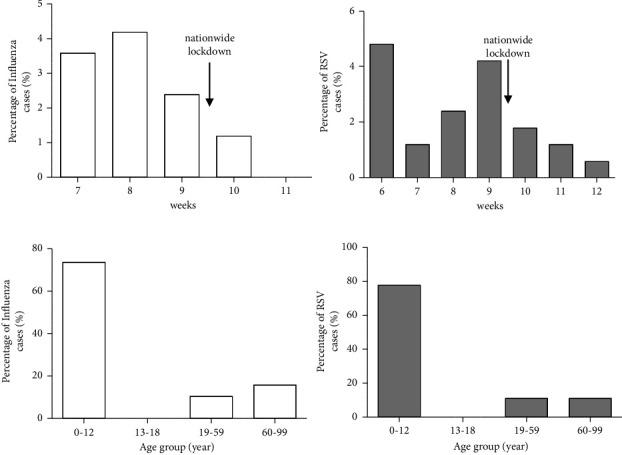
(a) Laboratory-detected cases of influenza A/B and RSV during the 2019/2020 season at our center. Vertical bars show percentage of INF-A/B and RSV positive specimens identified by week 6–7/2020 to 11–12/2020 (b) Distribution of INF-A/B and RSV cases during 2019/2020 season according to age groups.

We retrieved respiratory virus PCR data collected on our site during the 2019/2020 season, from week 46/2019 to week 12/2020. Overall, 19 out of 167 nasopharyngeal swabs (11.3%) were found positive for INF-A and/or INF-B viruses. The percentage of samples that tested positive for INF-A/B was highest in week 8/2020 (4.2%) and lowest in week 10/2020 (1.2%) (Figure 1(a)). The highest prevalence of INF-A/B infection was observed in the age group 0–12 years (73.6%) and the lowest in patients aged 13–18 years (0%) (Figure 1(b)). It should be noted that in week 10/2020 Italy started nationwide lockdown measures that included the closing of schools (March 9^th^, 2020). After the start of the lockdown (week 10), a sharp and significant decrease of INF-A/B cases compared to the preceding weeks was observed (2.39% vs 0.29%, *p* < 0.05). This is consistent with data from the national surveillance network InfluNet that showed the incidence of INF-A/B was below the baseline by week 12 in Italy, reporting the lowest INF-A/B prevalence ever recorded [4]. Furthermore, our data were in line with data reported by FluNet, the global web-based tool for influenza virological surveillance. FluNet showed an unusual absence of seasonal influenza activity in the temperate zone of the Northern Hemisphere compared to previous seasons, although sporadic influenza detections were reported in Southern Asia and tropical Africa where the transmission intensity remained at interseasonal levels across all countries during 2020/2021 [5–7]. During the season 2020/2021, the total seasonal prevalence of non-SARS-CoV-2 respiratory viruses for all specimens tested at IRCCS-SC was exceptionally low. In the whole of Italy, none of the respiratory samples tested positive for INF-A/B.

However, during the 2021/2022 season (from week 45/2021 to 4/2022), 10 influenza cases were detected. Eight out of 10 of the 2021/2022 samples were genotyped and identified as influenza type A; the subtype of seven of these was H3N2, and the other was H1N1. Two samples were not subtyped due to low viral loads. Interestingly, we observed one case of coinfection, detected during week 2 in a 37-year-old woman patient with a Ct for SARS-CoV-2 of 32 and 36 for INF-A. The genotype resulted to be A/H3N2.

InfluNet data recorded low levels of influenza virus transmission in Italy for the 2021/2022 season (up until the 4^th^ week of 2022). Overall, 55 INF-A and 5 INF-B strains have been identified since the start of the season, with the influenza A/H3N2 subtype as the most prevalent. Globally, very sporadic and localized influenza outbreaks were reported in the same period, with regional outbreaks of influenza A(H3N2) occurring in South and Southeast Asia, influenza B/Victoria in China, and influenza A(H1N1) in West Africa.

### 3.3. Prevalence of RSV-A/B

**Figure 2:**
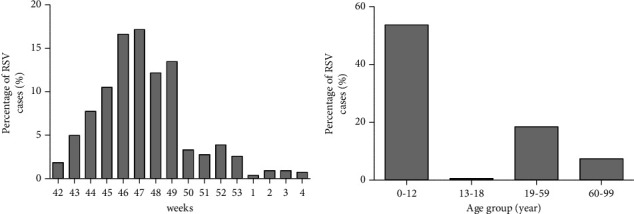
(a) Laboratory-detected cases of RSV during early 2021/2022 season at our center. Vertical bars show percentage of RSV positive specimens identified by week 42/2021 to 4/2022 (b) Distribution of RSV cases during 2021/2022 season according to age groups.

During the 2019/2020 season, we detected 17.3% RSV-A/B positive samples. RSV-A/B detection was most prevalent in the 0–12 years age group (77.7%). We also observed a decreasing trend in RSV-A/B cases after the implementation of the national lockdown in week 10 of 2020 (3.14% vs 0.89%, *p* < 0.05) (Figure 1(a)). However, as observed for influenza, the overall prevalence of RSV-A/B in the 2020/2021 season was atypically low. We did not detect any cases of RSV-A/B in our 0–12 years age group. Surprisingly, in the 2021/2022 season, after previous seasons of exceptionally low RSV circulation, a massive increase of RSV cases was observed from week 42 until week 49 of 2021, with a peak in week 47 (17.1%, Figure 2(a)), anticipating its circulation by about 1 month. RSV detection was higher in children aged 0–12 years (53.6%) and the lowest rate of infection was observed in age 13–18 (0.5%) years (Figure 2(b)). The number of RSV infections detected in the 2021/2022 season was significantly higher than that recorded during the 2019/2020 season (2.30% vs 14.29%, *p* < 0.01). Data from the USA and Australia showed an inter-seasonal increase in RSV infections that coincided with lifting lockdown restrictions [8, 9]. The resurgence of RSV detections probably resulted from circulation of the virus among a large susceptible pediatric population, and this led to a substantial increase in RSV-related hospitalizations [10]. These events illustrate the need for an effective prevention strategy for RSV in infants.

### 3.4. Study Limitations

This study has some limitations. First, because of the retrospective design of the study, important clinical characteristics were not recorded. Second, for the season 2019/2020, we performed less influenza testing compared to the 2020/2021 and 2021/2022 seasons, and this may lead to an underestimate of the prevalence of infection.

## 4. Conclusions

In summary, we reported the trend of influenza and RSV, during the last three flu seasons, in the course of the COVID-19 pandemic, in our reality. An exceptional low prevalence of INF-A/B was observed during season 2020/2021 and, curiously, an unprecedented surge in RSV cases was detected during the last flu season. This finding is related to mitigation strategies that were adopted to contain the COVID-19 pandemic. Our data strongly support the notion that the public health measures adopted by Italy and other countries to contain the SARS-CoV-2 outbreak modified the epidemiology and transmission of other respiratory viruses. To date, it is difficult to ascertain which NPI was most effective in limiting the spread of infections. Therefore, further investigations are needed to clarify the strength of associations between public health interventions and their impact on the basic reproductive number of respiratory viruses.

Systematic and continuous surveillance for non-SARS-CoV-2 respiratory viruses must be maintained in order to promptly prevent future epidemic surges of old or new respiratory viruses. In addition, efforts are needed to improve the RSV surveillance network.

## Figures and Tables

**Table 1:** Descriptive statistic of study population.

Demographics	Season 2019/2020		Season 2020/2021		Season 2021/2022	
	Counts (*n*)	Values (%)	Counts (n)	Values (%)	Counts (n)	Values (%)
Population	167		18112		14540	
Gender						
Female	77	46.10	9570	9570	7744	53.25
Male	90	53.89	8542	28.82	6796	46.74
Age (years)	Female	Male	Female	Male	Female	Male
Minimum	1.84	1.96	0.68	0.63	0.17	0.32
Mean	43.42	40.95	49.0	50.79	46.33	46.15
Lower 95% CI of mean	35.77	33.86	48.56	50.32	45.78	45.54
Upper 95% CI of mean	51.08	48.05	49.43	51.26	46.89	46.76
Median	50.84	50.14	48.7	52.9	46.74	48.12
Maximum	92.96	95.30	101.9	108.9	107	97.89
SD	33.73	33.89	21.51	22.08	24.95	25.63

## Data Availability

The data that support the findings of this study are available from the corresponding author upon reasonable request.
